# The Role of Surveillance Inspections in Reducing False-Positives of SARS-CoV-2 Omicron Variants during the COVID-19 Epidemic

**DOI:** 10.1155/2023/8508975

**Published:** 2023-04-19

**Authors:** Li-Li Liu, Yu-Hong Zheng, You-Quan Zhang, Jin-Piao Lin, Zhi-Lin Luo, Liu-Min Yu, Shi-Hua Gao, Falin Chen

**Affiliations:** ^1^Department of Center of Clinical Laboratory, Zhongshan Hospital of Xiamen University, School of Medicine, Xiamen University, Xiamen, China; ^2^Xiamen Clinical Laboratory Quality Control Center, Xiamen, China; ^3^Department of Laboratory, Fujian Medical University Cancer Hospital, Fujian Cancer Hospital, Fuzhou, China; ^4^Department of Laboratory, The Second Affiliated Hospital of Fujian University of Traditional Chinese Medicine, Quanzhou, China; ^5^Department of Laboratory, The First Affiliated Hospital of Fujian Medical University, Fuzhou, China; ^6^Department of Laboratory, The First Hospital of Putian City, Putian, China; ^7^Department of Laboratory, Affiliated Hospital of Putian University, Putian, China; ^8^The Affiliated Hospital of Fujian Medical University of First Hospital of Nanping, Nanping, China; ^9^Department of Clinical Laboratory, Fujian Provincial Hospital, Fuzhou, China

## Abstract

**Objective:**

This study aims to assess the effectiveness of surveillance inspections conducted by the provincial health committee in Quanzhou city during a COVID-19 outbreak in reducing false-positive results in SARS-CoV-2 RT-PCR assays.

**Method:**

The team conducted on-site inspections of laboratories that participated in mass screening, recording any violations of rules.

**Results:**

The positive cases in five rounds of mass screening were 23, 173, and 4 in Licheng District, Fengze District, and Luojang District, respectively. The false-positive rates in the five rounds of mass screening were 0.0099%, 0.0063%, 0.0018%, 0.0006%, and 0%, respectively. The study also recorded that the number of violations in the seven selected laboratories was 36, 68, 69, 42, 60, 54 and 47. The corresponding false-positive rates were 0.0012%, 0.0060%, 0.0082%, 0.0032%, 0.0060%, 0.0027%, and 0.0021%, respectively. The study found a positive correlation between false-positive rates and the number of violations (*r* = 0.905, *P*=0.005), and an inverse correlation between false-positive rates and the frequency of surveillance inspections (*r* = −0.950, *P* < 0.001).

**Conclusion:**

Daily surveillance inspection in laboratories can remind laboratories to strictly comply with standard procedures, focus on laboratory quality control, and reduce the occurrence of false-positive cases in SARS-CoV-2 nucleic acid tests to some extent. This study recommends that government decision-making departments establish policies and arrange experts to conduct daily surveillance inspections to improve laboratory quality control.

## 1. Introduction

COVID-19 is still in pandemic status and a public health emergency of international concern. The omicron variant is believed to have shorter serial interval (2.2–3.5 days) [1–3] and a shorter incubation period (2.9–3 days) than the delta variant [2, 4, 5]. Moreover, it has a higher secondary attack rate [6, 7] and has been proven to evade immune surveillance [4, 8–10]. Although omicron variant infection may be less likely to cause severe diseases and fatality, this specific variant can easily spread between individuals [11]. The impact of the omicron variant should not be ignored since it has a higher transmissibility, and if it spreads within a large population, a greater number of people could develop severe diseases. Additionally, some foreign countries have been observed in hospitalization rate and mortality related to the omicron variant [12]. Over the past two years, China has consistently implemented the dynamic zero-SARS-CoV-2 policy to prevent new outbreaks. However, in recent days, the domestic epidemic has significantly intensified, presenting complicated challenges to this policy. Laboratory tests for SARS-CoV-2 play a critical role in identifying the source of transmission and assisting government countermeasures. To further improve the quality of nucleic acid tests and identify potential issues, the Joint Prevention and Control Organization of the State Council has dispatched surveillance inspection teams to provinces experiencing outbreaks, to conduct problem-oriented surveillance inspections.

On March 13, 2022, nine people tested positive for COVID-19 during routine screening of key groups of people in Fengze District, Quanzhou City. All of the positive cases were hotel staff working for the same hotel. Genome sequencing of the positive samples revealed a sublineage that had evolved from the Omicron BA.2 mutant, and no highly homogeneous sequences were found when compared to other known sequences identified in China. In response, the provincial health committee dispatched a surveillance inspection team to high-risk areas of Quanzhou City. The team conducted surveillance inspections on compliance with operation manuals, personal protection, quality control, and cross contamination. The aim of these inspections was to improve quality control and eliminate the possibility of issuing inaccurate reports. On the fourth day after the deployment of the surveillance inspection team, 105 sample tubes tested positive, and on the fifth day, 159 sample tubes tested positive. The question remains whether there were any false-positive events in the reports. A review of 37 external quality assessments found that the false-positive rate in detecting RNA viruses ranged from 0.6% to 8.1% [13]. A false-positive result can have a negative impact on a patient's mental and physical health, and misdiagnosis can lead to mismanagement of medical resources. To the best of our knowledge, there has been no report on the evaluation of surveillance inspections on SARS-CoV-2 nucleic acid tests. This study aims to assess whether surveillance inspections can reduce false-positive events.

## 2. Materials and Methods

### 2.1. Study Population

From March 13, 2022, to April 6, 2022, PCR was used to perform the SARS-CoV-2 nucleic acid test on individual samples from 6.676 million subjects. A false-positive result was defined as a sample that tested positive in the first test but was found to be negative upon immediate retesting of the same fresh sample and testing in another laboratory. A total of 1.345 million people were screened in Licheng District, Fengze District, and Luojang District. The remaining 5.331 million people in Shishi City, Jinjiang City, Nanan City, Huian County, Quanzhou Economic and Technological Development Area, and Taishang Investment Area were also tested. In total, the entire city underwent five rounds of mass screening for COVID-19 between March 20, 2022, and March 28, 2022. During each round of mass screening, seven laboratories located in Licheng District, Fengze District, and Luojang District tested an average of 1,663,000 samples. Given the sample size and the geographic importance of these laboratories, the provincial surveillance inspection team conducted several rounds of inspections.

### 2.2. Inspection Approaches

The national molecular laboratory adopted the national standard “Medical institutions Novel Coronavirus nucleic acid Detection Manual” which is the national standard for carrying out novel coronavirus nucleic acid detection, and Quanzhou followed suit. The inspection list of supervising and testing experts was also based on this national standard. All nine supervisors from the province have held senior titles and have been engaged in molecular diagnosis for more than 10 years. The surveillance inspection team consisted of nine specialists who adopted a “circuit and on-site rotation” approach to conduct surveillance inspections in 26 laboratories. These laboratories had a total workload of 150 rounds of mass screening (an average of six rounds of tests for each laboratory) within 27 days.

The nine specialists employed a unified “Key Points for Quality Control Inspectors of SARS-CoV-2 Nucleic Acid Tests” as the inspection tool, and they recorded any violations of rules and sent to the provincial health committee. Laboratories were inspected on a daily basis. Information on positive and false-positive results during the five rounds of mass screening from March 20 to 28 was collected from seven laboratories out of 26 laboratories. Laboratory one, laboratory three, and laboratory four are located in Licheng District, laboratory two and laboratory five are located in Fengze District, and laboratory six and laboratory seven are located in Luojang District.

### 2.3. Positive Criteria and False-Positive Criteria

To be confirmed as positive cases, laboratory results must meet one of the two conditions. The first condition is that the real-time fluorescent RT-PCR test results of both ORF1 and N target genes of SARS-CoV-2 in the same sample are positive. If only one target gene tests positive, the sample must be retested or resampled for review. The second condition is that if the real-time fluorescence RT-PCR test results of a single target gene are positive in two samples of the same type, or if the test results of a single target gene are positive in two different types of samples taken at the same time, then the sample can be considered positive. For a positive sample, one to two more sensitive and amplified nucleic acid test reagents in different regions are used to retest the original sample.

If a positive sample from the same laboratory is retested by the Chinese Center for Disease Control and Prevention (CDC) and the result is positive, it is considered a true positive. If the retest is negative or the resampling and re-examination is negative, the sample is considered a false positive according to local policy. When a positive sample from the same laboratory is negative after CDC resampling, it is also considered a false positive. Samples with nucleic acid detection Ct value ≤ 32 undergo whole-genome sequencing by the CDC.

### 2.4. Statistical Analysis

Statistical analyses were conducted using SPSS version 20.0 (SPSS, Chicago, IL, USA). The normal distribution of continuous variables was examined using the Shapiro–Wilk test. The *Ct* values of the *ORF1* and *N* genes conformed to a normal distribution, and a *t*-test was employed to compare the groups. The chi-squared test was conducted to identify significant differences in qualitative variables across the groups. Spearman's rank correlation was used to analyze the correlations between the number of violations and the incidence of false-positive results, as well as the number of surveillance inspections and the incidence of false-positive results. A two-sided *P* value < 0.05 was considered statistically significant.

## 3. Results

### 3.1. Demographic Characteristics

According to the information from the local statistical network, the number of individuals under 18 years old in high-risk areas, specifically Licheng District, Fengze District, and Luojang District, was 95,289, 183,158, and 61,118, respectively. The number of individuals above 18 years old in these areas was 32,711, 517,842, and 18,688, respectively. With regards to sex distribution, the male populations were 208,067, 336,136, and 128,499, respectively, while the female populations were 219,933, 364,864, and 119,501, respectively.

During the five rounds of mass screening between March 20 and March 28, there were 23, 173 and 4 COVID-19 patients in Licheng District, Fengze District, and Luojang District, respectively (see Table 1).

### 3.2. False-Positive Events in SARS-CoV-2 Nucleic Acid Tests

**Figure 1:**
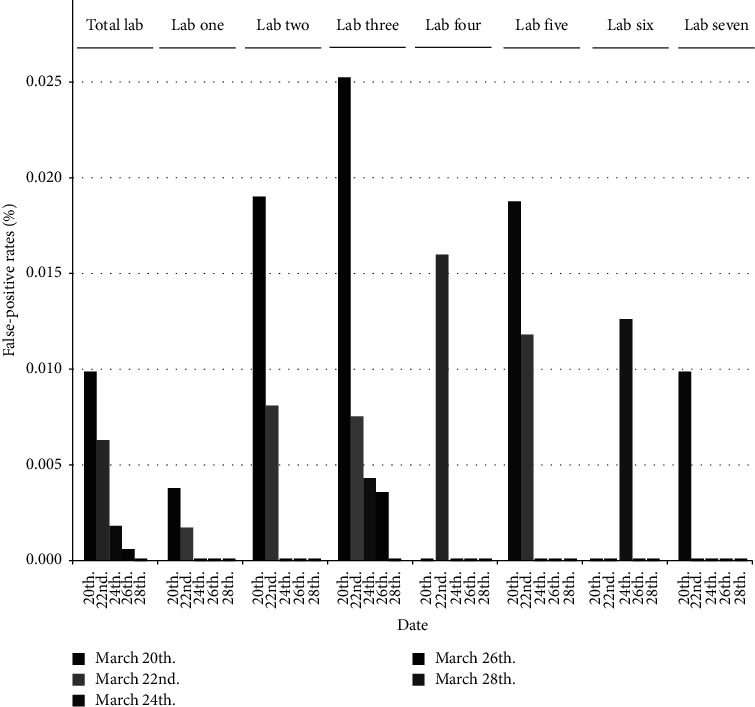
False-positive rates of SARS-CoV-2 detection at medical institutions.

During the five rounds of mass screening between 20 March and 28 March, the false-positive rates of SARS-CoV-2 Omicron variants in Licheng District, Fengze District, and Luojang District were 0.00028%, 0.00031%, and 0.00008%, respectively. The highest false-positive rate was observed in Fengze District. Seven laboratories were selected for further analyses. Laboratory one had false-positive rates of 0.004% ± 0.003% and 0.002% ± 0.002% on March 20 and March 22, respectively. During the same period, Laboratory two had false-positive rates of 0.019% ± 0.008% and 0.008% ± 0.006%. Laboratory three had false-positive rates of 0.025% ± 0.010%, 0.008% ± 0.005%, 0.004% ± 0.004%, and 0.004% ± 0.004% on March 20, March 22, March 24, and March 26, respectively, and had the highest absolute number and rate of false-positive events. Laboratory four had false-positive events only on 22 March, with a rate of 0.0016% ± 0.007%. For Laboratory five, false-positive rates of 0.019% ± 0.013% and 0.012% ± 0.012% were detected on 20 March and 22 March, respectively. False-positive events in Laboratory six were found only on 24 March, with a rate of 0.013% ± 0.009%, and similarly, false-positive events in Laboratory seven were observed only on March 20, with a rate of 0.010% ± 0.010%. The overall false-positive rates of SARS-CoV-2 Omicron variants in the five rounds of mass screening showed a gradual decreasing trend, with rates of 0.0099% ± 0.0024%, 0.0063% ± 0.0019%, 0.0018% ± 0.0011%, 0.0006% ± 0.0006%, and 0.0000% ± 0.0000% for each round, respectively (see Figure 1).

The false-positive rate of SARS-CoV-2 nucleic acid detection in seven laboratories decreased as the duration of the supervisions was extended. Laboratory one, two, and five had false-positive results for the SARS-CoV-2 nucleic acid test during the initial inspection, and the false-positive rate on March 24 was lower than that on March 22. On March 20, Laboratory three had the highest false-positive rate among the seven laboratories, and the false-positive rates for the four subsequent supervisions gradually declined. Only one false-positive test for SARS-CoV-2 nucleic acid was detected in Laboratory four, six, and seven, with false-positive rates of 0.0016% ± 0.007% on March 22, 0.013% ± 0.009% on March 24, and 0.010% ± 0.010% on March 20, respectively.

### 3.3. Comparison of the Results of the ORF1 and N Genes between True-Positive and False-Positive Samples

A comparison of the *Ct* values of the *ORF1* and *N* genes between true-positive and false-positive samples revealed that the *Ct* values of the *ORF1* and *N* genes in true-positive results were 29.11 ± 4.63 and 29.21 ± 4.55, respectively. In contrast, the *Ct* values in false-positive results were 35.67 ± 3.12 and 34.99 ± 2.96, respectively (refer to Table 2). Statistical analyses demonstrated that the *Ct* values of the *ORF1* and *N* genes in the true-positive results were significantly lower than those of the false-positive results (*t* = −4.107, *P* < 0.001, *t* = −3.522, *P* < 0.001).

### 3.4. Analysis of the Violations of Rules in the Seven Laboratories

Misconducts that could lead to inaccurate laboratory testing were observed during the five rounds of surveillance inspections conducted between March 20 and March 28. The rules violated by the seven laboratories are presented in Table 3. As seen in the table, the most frequently violated rules were A-2, B-3, B-4, D-4, E-1, and F, which were all violated by four of the laboratories.

### 3.5. The Association between Violations of Rules and False-Positive Events

**Figure 2:**
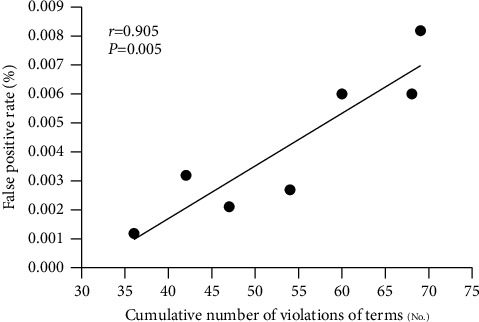
Association between violations of rules and false-positive events.

The observed incidences of rule violations in the seven laboratories (Lab 1 to Lab 7) were 36, 68, 69, 42, 60, 54, and 47, respectively, in the period between March 20 and March 28. The false-positive rates of SARS-CoV-2 Omicron variants in the inspected laboratories were 0.0012% ± 0.0007%, 0.0060% ± 0.0021%, 0.0082% ± 0.0026%, 0.0032% ± 0.0014%, 0.0060% ± 0.0034%, 0.0027% ± 0.0019%, and 0.0021% ± 0.0021%, respectively. There was a positive correlation between the false-positive rate of SARS-CoV-2 Omicron variants and rule violations (*r* = 0.905, *P*=0.005, Figure 2).

Spearman's rank correlation was used to analyze the correlation between the false-positive rate and violation of rules. The false-positive rate of SARS-CoV-2 Omicron variants and violation of rules were positively correlated (*r* = 0.905, *P*=0.005).

### 3.6. The Effect of Inspections on False-Positive Results

**Figure 3:**
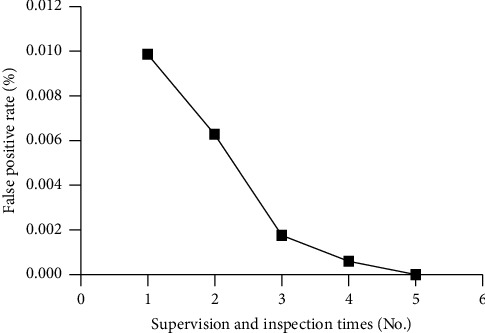
Association between the number of inspections and false-positive rate.

In the first round of surveillance inspection, the false-positive rate of SARS-CoV-2 Omicron variants was 0.0099% ± 0.0024%; the rate decreased to 0.0063% ± 0.0019% in the second round, and continued to decrease as the inspections continued. The rate was 0.0018% ± 0.0011% in the third round, 0.0006% ± 0.0006% in the fourth round, and finally reached 0 in the fifth round. The false-positive rate of SARS-CoV-2 Omicron variants and the number of inspections were inversely correlated (*r* = −0.950, *P* < 0.001, Figure 3).

Spearman rank correlation was used to analyze the correlation between the false-positive rate and supervision and inspection times. The false-positive rate of SARS-CoV-2 Omicron variants and the number of inspections were inversely correlated (*r* = −0.950, *P* < 0.001).

## 4. Discussion

On March 13, a surveillance inspection team was dispatched to the high-risk area and noticed an increase in positive COVID-19 cases. The nucleic acid test is the primary method used for diagnosing COVID-19, and it provides direct evidence [14, 15]. However, if a laboratory operates at or above its full capacity, it may still function at the same quality as usual, or false-positive results may occur. During the surveillance inspection from March 20 to March 28, almost every laboratory involved in the mass screening produced false-positive results. The positive rates in the three high-risk areas were highest in Fengze District, followed by Licheng District and Luoyang District. Laboratory three had the highest number of false-positive samples as well as the highest false-positive rate. The main reasons behind these high false-positive events were manual operational errors. Laboratory three had two operating sections: a *P*2 biosafety laboratory in a fixed location operated by professional medical laboratory technicians and a mobile cabin laboratory operated by scientific researchers and backups from other health institutions. Therefore, inadequate training may have led to quality issues. False-positive results, in general, were primarily attributed to mislabelling and contamination [16]. Mislabelling and contamination were the primary causes of false-positive results, such as contamination caused by primers or probes in a commercialized PCR kit for SARS-CoV-2 [17].

During our surveillance inspection, we identified several factors that contributed to false-positive results. The most common factor was contamination between samples or with a positive control. Inadequate sterilization of sample tubes or failure to sanitize hands or replace gloves after handling a positive control can lead to cross-contamination. When using small pipette tip, inserting the pipette too deeply into the sample tube can contaminate the pipette and the sample. Vigorous vortexing of the sample without sufficient standing time can cause cross-contamination through aerosols. These operational details are consistent with findings reported in the literature [18]. A second common source of false-positive results is amplification products. Montgomery et al. also found that the cDNA of SARS-CoV-2 can cause environmental contamination [19]. Contamination can occur if microplates are not sealed after amplification or if the seal bag is not sterilized; steam sterilization of the PCR product can then cause product contamination. Failure to follow the rule of moving only in a single direction can result in contamination in multiple areas. Ultimately, the analysis of results can also contribute to false-positive results. The default threshold set by the manufacturer's software may cause false-positives if there are one or more wells with large fluctuations in the baseline; even a bubble can cause a false-positive result.

If there are more than a certain number of positive results in a laboratory, the internal quality control should be analyzed, and the population should also be examined. If the population is the same as that previously tested and the amplification curve is highly similar, the possibility of contamination should be considered. To prevent the occurrence of false-positive results, the first priority is to perform testing in accordance with the standard operation procedures. Second, surveillance of contamination should be conducted. Specifically, four spots in a biosafety cabinet, four random spots in a sample rack, extraction instruments, cap-removal instruments, the body of a pipette where the hand grips, and the door handle of a freezer should be regularly tested to prevent cross-contamination between samples. To detect aerosol contamination, sample tubes with RNAse- and DNAse-free water should be placed, with the lid open, on benches in the reagent and sample preparation areas, inside biosafety cabinets and extraction instruments, and on the benches with the amplification instruments. The sample tubes with water should be left open for 6 to 8 hours during sample testing and no less than 16 hours during idle time. Subsequently, the sample tubes with water should be vortexed and tested. If sample contamination or aerosol contamination is detected, testing should be suspended, and the laboratory should be completely ventilated and cleaned. The monitoring process should be repeated until the results are negative. Third, the confirmation of results and records proper record-keeping should be ensured. The present study revealed that the *Ct* values of the *ORF1* and *N* genes in false-positive samples were significantly higher than those of true-positive samples. The results of this study suggest if a sample has a *Ct* value for the *ORF1* gene greater than 35.67 and a *Ct* value for the *N* gene greater than 34.99, the results are questionable, especially when a sudden increase in the number of positive cases is observed in a low-risk area for COVID-19. Falasca et al. also suggested that detection of the *N2* gene at high *Ct* needs to be interpreted with caution [20].

Through our surveillance inspection, we found that the more laboratories violated the rules, the higher the false-positive rate was. The supervising experts communicated with the laboratory staff on-site and provided instruction and demonstrated correct operation. With the increases in the numbers of supervisions and inspections, the incidence of false-positives in the laboratory gradually decreased.

## 5. Conclusion

Continued surveillance inspections can improve the quality control of SARS-CoV-2 RT‒PCR assays in laboratories, uncover details of irregularities in laboratory operations, and help identify prevention measures targeting laboratory contamination. This study analyzed the possible reasons for false-positive results in the preanalytical, analytical, and postanalytical phases of nucleic acid tests. The insights provided may shed light on operators of SARS-CoV-2 RT-PCR tests reduce the incidence of these false-positive results, as well as unnecessary quarantine and contact tracing. This study advocates that government decision-making departments formulate policies and arrange for experts to carry out daily surveillance inspections, which is an effective way to improve laboratory quality control.

## Figures and Tables

**Table 1:** Demographic characteristics of subjects.

Variable	
Age (years)	
<18 years (no.)	
Population of Li-district	95289
Population of Feng-district	183158
Population of Luo-district	61118
≥18 years (no.)	
Population of Li-district	332711
Population of Feng-district	517842
Population of Luo-district	186882
Sex	
Male (no.)	
Population of Li-district	208067
Population of Feng-district	336136
Population of Luo-district	128499
Female (no.)	
Population of Li-district	219933
Population of Feng-district	364864
Population of Luo-district	119501
Administrative area	
Total population of Li-district	428000
Number of COVID-19 patients in Li-district on March 20–28	23
Total population of Feng-district	701000
Number of COVID-19 patients in Feng-district on March 20–28	173
Total population of Luo-district	248000
Number of COVID-19 patients in Luo-district on March 20–28	4

Abbreviation: no., number.

**Table 2:** Comparison of ORF1 and N gene results between true-positive and false-positive samples.

	The number of true-positivesamples	True-positive results (Ct, mean ± SD)	The number of false-positivesamples	False-positive results (Ct, mean ± SD)	*t* value	*P* value
ORF1 genes	30	29.11 ± 4.63	80	35.67 ± 3.12	−4.107	<0.001
N genes	30	29.21 ± 4.55	80	34.99 ± 2.96	−3.522	<0.001

ORF1: open reading frame 1, and N: nucleocapsid protein, Ct: cycle threshold, SD: standard deviations.

**Table 3:** Distribution of violations of rules in the seven laboratories.

Rules	Number of laboratories that violate this rule	Proportion of laboratories that violate this rule (%)	*χ* ^2^	*P*
A-1 Proper donning and doffing of PPE	3	43	19.529	0.423
A-2 Proper decontamination after contacting positive control materials or samples	4	57		
B-1 Airflow control of the laboratory	3	43		
B-2 Regular surveillance of the environment	3	43		
B-3 Maintenance of a sufficient supply and proper use of consumables for sanitation, sterilization and protection	4	57		
B-4 Proper disposal of medical waste	4	57		
C-1 Laboratories should be equipped with instruments approved to conduct nucleic acid testing for SARS-CoV-2	1	14		
C-2 Using DNase- and RNase-free consumables	1	14		
C-3 Validation of reagents and instruments used for extraction and amplification	1	14		
D-1 Using amplification reagents in accordance with manufacturers' operating manuals	3	43		
D-2 Separation of amplification reagents and samples	1	14		
D-3 Products must be sealed after extraction	1	14		
D-4 Sterilization of receptacles for sample tubes	4	57		
E-1 Prevention measures to avoid contamination inside a biosafety cabinet	4	57		
E-2 Adding template to a reaction system inside a biosafety cabinet	1	14		
E-3 Prevention measures for adding samples and postextraction procedures	2	29		
E-4 Proper decontamination of laboratory benches soiled by pipette tips	1	14		
E-5 Inspection of liquid volumes and bubbles in 8-tube strips before extraction	1	14		
F Proper execution of internal quality control	4	57		
G-1 Proper review process of test results	3	43		
G-2 Proper record keeping	3	43		

## Data Availability

Data used to support the findings of this study are available from the author upon request due to ethical issues.

## Abbreviations:

lab: Laboratory
20th: Twentieth
22nd: Twenty-second
24th: Twenty-fourth
26th: Twenty-sixth
28th: Twenty-eighth.
